# Supplementary material for: A high-resolution mRNA expression time course of embryonic development in zebrafish
Source: eLife. 2017 Nov 16;6:e30860. doi: 10.7554/eLife.30860 (PMC5690287; doi:10.7554/eLife.30860)
Supplement: Supplementary file 6. [file elife-30860-supp6.zip › biolayout-clusters-files/Cluster099-genes.html]

Cluster099


# Cluster099: Genes

| Ensembl ID | Gene Name | Chr | Start | End | Biotype |
| --- | --- | --- | --- | --- | --- |
| ENSDARG00000102959 | ZNF497 (1 of many).1 | 21 | 2151292 | 2206768 | protein\_coding |
| ENSDARG00000054374 | alg13 | 21 | 34199002 | 34227294 | protein\_coding |
| ENSDARG00000056559 | ccz1 | 12 | 17535350 | 17564530 | protein\_coding |
| ENSDARG00000022690 | chic2 | 1 | 13719585 | 13729922 | protein\_coding |
| ENSDARG00000103933 | fancm | 17 | 1465227 | 1476786 | protein\_coding |
| ENSDARG00000016474 | galcb | 17 | 18869801 | 18878279 | protein\_coding |
| ENSDARG00000025949 | irak1 | 8 | 7552364 | 7584969 | protein\_coding |
| ENSDARG00000029511 | kcns3b | 20 | 14218278 | 14222316 | protein\_coding |
| ENSDARG00000093597 | oip5 | 13 | 33126397 | 33129349 | protein\_coding |
| ENSDARG00000052617 | raver1 | 3 | 48607272 | 48634672 | protein\_coding |
| ENSDARG00000094737 | si:ch211-12e13.12 | 5 | 8719413 | 8732484 | protein\_coding |
| ENSDARG00000092800 | si:ch73-77n15.1 | 10 | 23964326 | 24079743 | antisense |
| ENSDARG00000091212 | si:dkey-192p21.6 | 13 | 25511600 | 25590457 | protein\_coding |
